# Perceiving action boundaries for overhead reaching in a height-related situation

**DOI:** 10.3758/s13414-021-02293-2

**Published:** 2021-03-29

**Authors:** Lisa P. Y. Lin, Sally A. Linkenauger

**Affiliations:** Department of Psychology, Fylde College, Lancaster University, Bailrigg, Lancaster, LA1 4YF UK

**Keywords:** Perception, Action boundaries, Perceptual-motor calibration

## Abstract

To successfully interact within our environment, individuals need to learn the maximum extent (or minimum) over which they can perform actions, popularly referred to as action boundaries. Because people learn such boundaries over time from perceptual motor feedback across different contexts, both environmental and physiological, the information upon which action boundaries are based must inherently be characterised by variability. With respect to reaching, recent work suggests that regardless of the type of variability present in their perceptual-motor experience, individuals favoured a liberal action boundary for horizontal reaching. However, the ways in which action boundaries are determined following perceptual-motor variability could also vary depending on the environmental context as well as the type of reach employed. The present research aimed to established whether the perceptual system utilises the same strategy for all types of reaches over different contexts. Participants estimated their overhead reachability following experience reaching with either a long or a short virtual arm, or a virtual arm that varied in length – while standing on the edge of a rooftop or standing on the ground. Results indicated that while similar strategies were used to determine action boundaries in both height- and non-height-related context, participants were significantly more conservative with their reachability estimates in the height-related context. Participants were sensitive to the probabilistic information associated with different arm’s reach they have experienced during the calibration phase, and used a weighted average of reaching experience to determine their action boundary under conditions of uncertainty.

## Introduction

To select and modify movement plans adaptively, the perceiver needs to be sensitive to their action boundaries. Action boundary is the critical point or limit that separates possible actions from impossible actions, and actions are only possible when they are within one’s action boundary (Fajen, 2005). Consequently, action boundaries vary depending on the individual, for example, an object that affords reaching for an adult may not afford the same for a child, due to the differences in their body morphology and motor abilities.

People have been shown to be highly sensitive to the boundaries of their action capabilities (Carello et al., 1989; Ishak et al., 2014; Warren, 1984; Warren & Whang, 1987). Additionally, people could rapidly recalibrate to a new action boundary and modify their affordance judgements following changes in their body dimensions and action capabilities. Such examples include updating their judgements of passability when fitting one’s hand through an opening when their hand width has been enlarged by a prosthesis attached to their hand (Ishak et al., 2008) and passing through doorways while wearing a different sized artificial belly (Franchak & Adolph, 2014). Individuals also adjust their maximum sitting or stepping height judgement while wearing platform shoes/blocks under their feet (Hirose & Nishio, 2001; Mark, 1987) and decrease their jumping ability judgements when wearing ankle weights (Lessard et al., 2009).

Action boundaries change over the course of lifetime due to variations in one’s action capabilities caused by physical or physiological changes in one’s body associated with natural processes. However, much like our environments, our bodies and our action capabilities are not stagnant. Variability is always present when we navigate our surroundings, and studies have shown that individuals account for their own movement variability when making action boundary judgements. For instance, children and older adults have been shown to leave a greater margin of safety when judging whether an aperture affords passing, and they also rotate their shoulders to a greater extent for a given aperture size compared to younger adults (Hackney & Cinelli, 2011; Wilmut & Barnett, 2010, 2011). These group differences suggest that individuals take into account their action capabilities and movement variability by making more conservative action boundary judgements. Additionally, factors such as injuries, flexibility, anxiety or fatigue can also lead to changes in the body, and in turn fluctuations in action capabilities (Franchak & Adolph, 2014a; Konczak et al., 1992; Pijpers et al., 2006; Pijpers et al., 2007). Hence, regardless of how consistent an action’s outcome may seem, the perceptual motor information specifying action boundaries is always characterised by some amount of variability. As a result, the perceptual system must select an action boundary from a variety of perceptual motor experiences that conflict in terms of their indication of the perceiver’s maximum reachability.

One such solution that the perceptual system could employ would be to select action boundary size using something akin to a weighted average, in which prior perceptual motor experiences are combined on the basis of their relative likelihood to identify the most statistically likely outcome (Deneve & Pouget, 2004; Körding & Wolpert, 2006). To determine the appropriate action boundary from the most likely outcome when considering all similar perceptual motor experiences, one could assign weighting to action boundaries based on the probabilistic information associated with each action boundary they have experienced during reaching experience. For instance, consider an individual who has experienced two different action boundary size (large and small) during their reaching experience, in which they experienced the large action boundary half of the time and the small action boundary half of the time. Given that they have experienced both action boundaries with equal probability, they could then take the average of the action boundary experienced – which would be similar to the mean. Alternatively, if they have experienced the large action boundary 75% of the time, and 25% of the time they experienced the small action boundary, then more weight would be assigned to the large action boundary as it was encountered more often than the other action boundaries. The selected action boundary would be closer to the large action boundary (but not as large) they have experienced during the reaching experience, because it is more statistically likely than a smaller one. Hence, by incorporating probabilistic information in the selection of action boundary, we would expect individuals’ action boundary estimates to reflect a systematic shift in size depending on the weighting attributed to each action boundary experienced.

While this method may allow for an optimising approach to determining action boundaries, it does not come without a cost. Such information processing, i.e., taking into account all experiences and weighting them with respect to their reliability, incurs considerable temporal and energetic costs, and the brain is the most energy consuming organ in the human body (Clarke & Sokoloff, 1999; Niven & Laughlin, 2008). Evolutionary approaches have characterised the optimising processes underlying such computations as inefficient given that human cognitive capacities are necessarily limited, and some have argued that perceptual systems function to satisfice and produce adaptive behaviours rather than to optimise (Hoffman et al., 2015). Heuristics provide satisficing solutions that are time and effort efficient (i.e., require less computation), and heuristics produce comparable and more energetically adaptive solutions than more complex computations in real-world situations (Gigerenzer & Gaissmaier, 2011; Martignon, 2001). Nevertheless, this may also depend on the situation, and it is possible that more deliberated computation may be required in situations where the stakes are high.

Hence, the perceptual system could use heuristics for a fast and efficient evaluation, by examining fewer alternatives and adopting a single action boundary that doesn’t vary drastically regardless of the probabilistic information associated with each possible action boundary, and nonetheless achieve satisfactory performance. One possible heuristic is that the perceptual system could select the action boundary using the most liberal-sized action boundary experienced. This method is akin to signal detection theory – in a situation that requires you to reach a target, if you think that you could possibly reach the target, then you would always attempt to do so (e.g. Green & Swets, 1966; Swets et al., 1961). Consequently, in the event that the action capabilities of an individual fluctuate constantly, attempting the action using the most liberal-sized action boundary experienced would result in the highest number of successful attempts. However, this option would only be beneficial to the individual in the absence of consequences associated with a failed action, because it would lead them to fail more often as well. Alternatively, individuals could use the most conservative-sized action boundary experienced regardless of the variability. This option would be in the perceiver’s best interest especially when making motor decisions in situations in which motor errors are associated with negative consequences. However, this method would also result in the smallest number of successful attempts.

Recent studies have investigated participants’ judgements of action boundaries for reaching following changes in their action capabilities in a virtual environment. Lin et al. (2020) had participants estimate their action boundary for horizontal reaching following calibration to a long virtual arm, a short virtual arm or a variable virtual arm that varied randomly but in equal frequency between a long, medium and short virtual arm. In the following experiments, the design was the same, except that in the variable condition, the frequency of the virtual arm lengths varied systematically in that they were greatly weighted towards the long virtual arm or the short virtual arm. Across three experiments, participants recalibrated to a new action boundary that was consistent with their reaching experience and estimated their reachability to be farther in the consistent long virtual arm conditions than in the consistent short virtual arm conditions. Interestingly, findings demonstrated that the pattern of results was similar regardless of whether participants experienced all reaches with equal probability or whether their perceptual motor experience in the variable conditions was systematically weighted towards the long virtual arm or the short virtual arm. Participants estimated their reachability in the variable condition more similarly to when they were calibrated only with a long virtual arm’s reach. This finding suggests that individuals may have selected an action boundary using heuristics and employed a liberal approach when estimating action boundaries in the event of perceptual motor variability.

However, Lin et al.’s (2020) results may be due to the specific action being performed and the context in which the action is performed. Consider overhead reaching, in contrast to horizontal reaching. Reaching vertically is kinematically different from reaching horizontally, not only is the actor’s overall postural configuration different, the perceiver must also maintain their balance while executing the reach. Hence, selecting the action boundary using the most liberal reach experienced may not be the most appropriate strategy as a failed liberal reach may impair their ability to maintain balance and result in falling. Previous research has shown that individuals tend to overestimate their reachability, and they perceived targets that are out of reach to be reachable (Fischer, 2000; Rochat & Wraga, 1997). However, individuals were found to be more conservative with their estimates or even underestimate their reachability when executing reaches that would shift their centre of mass beyond the base of support of their feet, such as reaching for high objects while standing or reaching while bending at the hip (Carello et al., 1989; Robinovitch, 1998). Hence, perceived action consequences associated with postural stability may lead to more conservative action boundary estimation. The perceptual system could change its strategy in the way action boundaries are determined following perceptual-motor variability depending on the consequences of failing. If this is the case, then individuals would be more conservative with their action boundary when the reaching task requires greater postural stability demands.

Similarly, with respect to context, in Lin et al. (2020), failed action was not associated with any negative consequences. Hence, by selecting the liberal action boundary, participants were likely trying to maximise their probability of success while disregarding their probability of failure. However, in contexts where there are penalties for selecting the inappropriate action boundary, individuals may be more conservative with their judgements. For instance, younger adults, older adults and infants have been shown to make more conservative motor decisions when navigating through doorways when the penalty associated with motor decision errors was falling in comparison to when the penalty for error was to become wedged (Comalli et al., 2013; Franchak & Adolph, 2012). Therefore, we suspect that the context in which the action occurs, and the resulting consequences associated with failed action, would influence how individuals account for perceptual-motor variability when determining their action boundaries.

Nevertheless, these attributes may be difficult to investigate in the real world, due to the consistency of individuals’ bodies and action capabilities, as well as the possibility of incurring risks or injuries to participants. However, by using virtual reality and motion-capture technology, we would be able to investigate these attributes in a safe yet realistic manner. Studies using virtual reality have found that individuals react to and interact with the virtual environment as if they were real and exhibited behavioural and physiological responses that are comparable to those occurring in the real world (Slater et al., 2006). In this set of studies, we have opted to use virtual height-related situations as a potential perceived risk or negative consequence associated with failed action. Fear of heights is one of the most common types of fears, and one of the earliest acquired ones (De Jongh et al., 2011). After a few weeks of self-generated locomotor experiences, 6-month-old infants show a wariness of heights and avoid the deep side of the visual cliff (Bertenthal et al., 1984; Gibson & Walk, 1960). Furthermore, height fear has been shown to influence visual perception, in which individuals with greater level of acrophobia perceived vertical extents to be higher (Stefanucci & Proffitt, 2009; Teachman et al., 2008). Virtual reality has also been used as a medium for exposure treatment for various types of phobias, including fear of heights. Individuals have reported physical symptoms of anxiety when in virtual height situations, and their fear of heights was reduced successfully after several sessions of virtual reality exposure (Regenbrecht et al., 1998; Rothbaum et al., 1995). Taken together, we believe that a virtual heights situation would allow us to examine whether individuals could associate negative action consequences with their selection of action boundaries under conditions of perceptual motor variability.

In a series of studies, we examined the effect of environmental context and the type of perceptual-motor variability in reaching experience on the perception of action boundaries for overhead reaching using virtual reality. Participants engaged in a calibration phase where they executed a series of reaches to targets of various heights with a long virtual arm, a short virtual arm or a virtual arm that varied in size randomly or systematically across reaching trials. Participants performed this calibration while standing on the edge of a tall building or standing on a horizontal ground plane. After the calibration phase, participants estimated their maximum reaching ability. We expected individuals to employ different strategies when determining their action boundaries in different environment contexts. It is possible that individuals are more deliberate/conservative in the height-related situation, and incorporate probabilistic information associated with the reach lengths they have experienced during the calibration phase into their action boundary judgement as a result of negative consequences. If so, their reachability estimates would likely reflect a systematic shift in size depending on the weighting attributed to each arm’s reach experienced, in that they would favour a more liberal size action boundary if they have experienced a long virtual arm’s reach more often than other reaches. In the non-height-related situation where failed action is not associated with negative consequences, individuals would adopt an action boundary size that does not vary drastically regardless of the probabilistic information associated with each possible action boundary.

## Experiment 1

In this experiment, we investigated the effect of random variability on the perception of action boundaries in a high-risk situation. In a virtual environment, participants estimated their maximum reachability after being calibrated with a long virtual arm, a short virtual arm or a virtual arm that varied in size randomly.

## Method

### Participants

G*Power software application (Faul, Erdfelder, Buchner & Lang, 2009) was used to perform an a priori power analysis to estimate sample sizes required to achieve adequate power. The required power was set at 1- β = .85, and the level of significance was kept at α = .05. We expected a medium effect size of .25 due to the novelty of the paradigm. Power analysis indicated that a sample of N = 15 would be sufficient to achieve a power of .85 and an alpha of .05. We have increased our sample size to a minimum 20 participants for all four experiments due to the possibility of technical failure with this type of equipment.

Twenty-one participants (15 females) between 18 and 29 years of age (Mage = 21.05 years, SDage = 2.64 years) were recruited from Lancaster University through opportunity sampling. All participants but two were right-handed. All participants had normal or corrected-to-normal vision. All participants provided informed consent. This study was approved by the ethics committee at Lancaster University.

### Stimuli and apparatus

The experiment was conducted in front of a table and a chair was placed in front of it. The chair was placed against the table and in front of the participants to minimise the risk of participants losing their balance; participants stood roughly 40 cm from the table. Participants wore an Oculus Rift CV1 head-mounted display (HMD) that displayed a stereoscopic image of the virtual environment with a resolution of 2,160 × 1,200 pixel and a frame rate of 90 Hz. The position of participants’ arms and hands were tracked using a Leap Motion hand-tracking sensor mounted on the front of the Oculus HMD. The leap motion fully animates the arm and individual finger movements in real time based on the movements of the user.

The experimental program and environment were created using Unity 3D© Gaming Engine with the Leap Motion plugin. For the virtual environment, a three-dimensional (3D) model of a city with skyscrapers was used. The virtual avatar was placed on the edge of the rooftop of one of the skyscrapers; a 3D model of a transport chopper was placed above the avatar, with a ladder that extended from the bottom of the chopper.

**Fig. 1:**
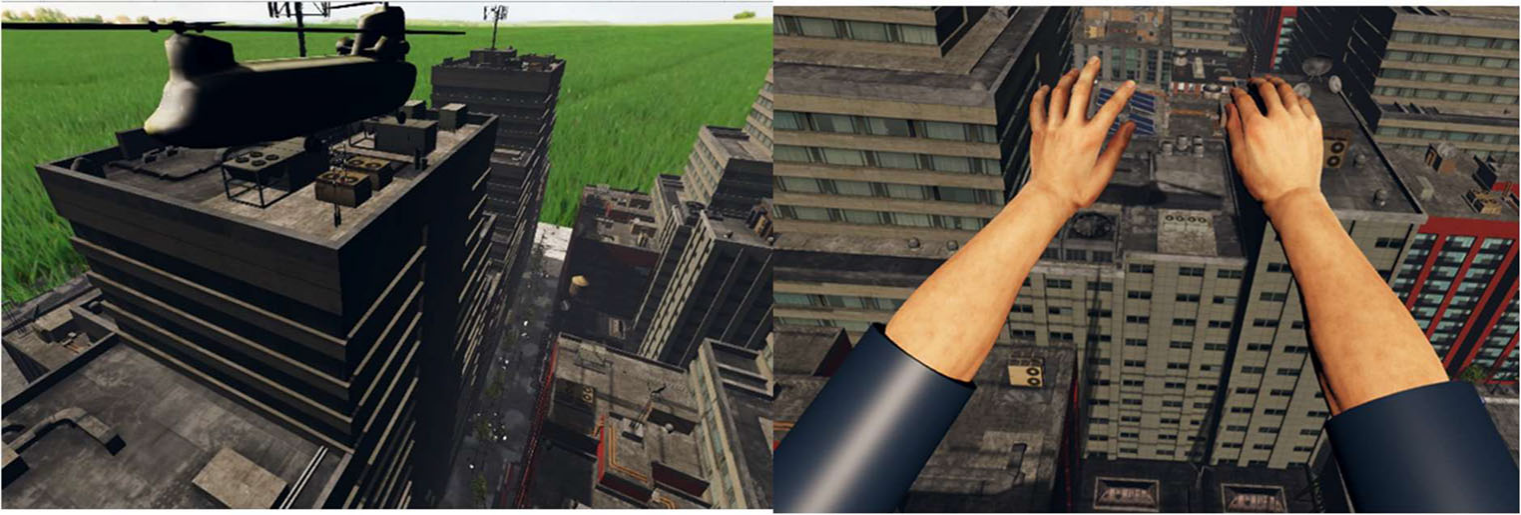
Left panel: Screenshot of the virtual environment showing the complete scene. Right panel: Image of what the participant would see from their perspective during the calibration trial

The 3D camera was placed at eye level enabling the participant to perceive the virtual environment in a first-person perspective, and the position of the 3D camera was consistent with participant’s physical eye-height. They were positioned in the virtual environment so that they were standing under the chopper and in front of the ladder (see Fig. 1). The movement of the participant’s head was tracked, and graphics were updated as the participant looked around in the virtual environment by moving their head. The movement and position of the participant’s tracked hands were mapped onto the virtual arm and hand, so that the movement of the virtual hand was congruent with the movement of the participant’s actual hand. The avatar hands that we used were taken from the realistic human hand models provided by the Leap Motion V2 SDK. Three different virtual arm sizes were used: the original arm model was used for the normal arm’s reach; the length of the original arm model was mapped onto the physical model derived from actual arm length of each participant. For the extended arm’s reach, the virtual arm was scaled as 50% longer than the original arm model, and for the constricted arm’s reach, the virtual arm was scaled as 50% shorter than the original arm model.

### Procedure

After providing informed consent, participants were asked to stand facing the table. They were given instructions for both the calibration and the estimation phases of the experiment. After donning the Oculus HMD, participants completed all three experimental conditions, and participants were randomly assigned to different orders of conditions. In the extended-reach condition, the virtual arm was 50% longer than the participant’s normal arm and was made to reach 50% farther than their physical reach. In the constricted reach condition, the virtual arm was limited to 50% of the participant’s physical reach with the arm being 50% shorter than the participant’s normal arm. In the variable reach condition, the virtual arm varied between the extended arm’s reach, the constricted arm’s reach, and the normal arm’s reach; participants experienced all reaches with equal probability (i.e., equal number of trials).

**Fig. 2:**
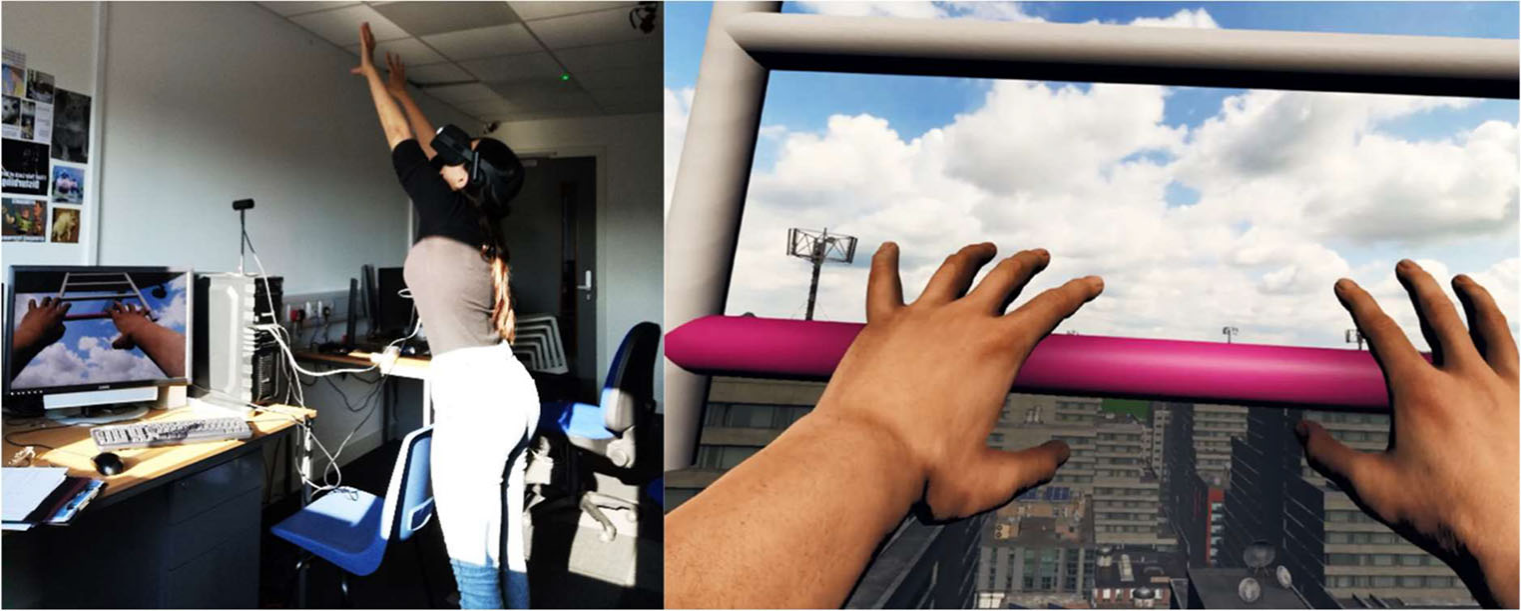
Left panel: Illustration of a participant completing a calibration trial. Right panel: Image of what the participant would see while completing the calibration phase

Each condition consisted of two parts: calibration and estimation. The calibration phase consisted of 48 trials in which a pink-coloured ladder rung was presented in front of the participant at various vertical heights. Participants were instructed to reach and grab the pink bar with their virtual hands. If the bar was too far or high for the participant to reach, they were instructed to point towards it instead (see Fig. 2). After they reached out and touched/pointed at the bar, the bar disappeared and another pink bar at a different location appeared. The bars were presented at one of the six vertical distance from the rooftop to which the participant was standing on (140, 160, 180, 200, 220, and 240 cm), for a total of six possible locations each presented eight times for a total of 48 trials, with the bar location being presented in randomised order.

Participants engaged in an estimation phase following each calibration phase. Prior to beginning the estimation phase, participants were told to estimate their reaching ability in the virtual environment. To prevent participants from counting and memorising the number of times they had pressed the arrow key, the experimenter would adjust the estimation bar for participants while looking away from the monitor as each trial began. The estimation phase consisted of 12 trials, the experimenter used the arrow keys to move the position of an orange-coloured bar (estimation bar), and participants were instructed to inform the experimenter when to stop so that the bar was just within their reach. The up-arrow key moved the estimation bar upwards and the down arrow key moved the bar downwards. Each button press moved the bar 5 cm upwards or downwards. During the estimation phase, the virtual hands were removed from the scene so that participants had no visual feedback about their arm length. For half of the trials, the estimation bar originated from 100 cm above the rooftop. In order to control for hysteresis – the phenomenon in which the individuals’ estimates are typically longer if the stimulus starts away from the perceiver and is moved toward the perceiver relative to when the stimulus starts close to the perceiver and is moved away. Hence for the other half of the trials, the estimation bar’s starting position was 280 cm above the rooftop. The bars thus either started below or above the participants, for a total of two locations each presented six times for a total of 12 trials. Participants were reminded that there was no right or wrong answer, and they could make as many fine adjustments as they needed until they were satisfied with their estimate of their reaching ability. Once they were satisfied with their estimate, the bar disappeared and the next trial began. To sum up, each participant completed three reaching conditions (extended, constricted, variable) in randomised order, and in each condition they completed a calibration phase consisting of 48 trials followed by an estimation phase consisting of 12 trials.

## Results

To account for the height of the building rooftop the participants were standing on, we have subtracted 64.228 from the raw reaching estimates. To analyse the influence of reaching condition on reachability estimates, where reachability was defined as the farthest extent to which participants estimated they could reach vertically, we employed a repeated-measures ANOVA with reaching condition (extended/ constricted/variable) as within-subjects variable and the estimated reachability as the dependent variable.

**Fig. 3:**
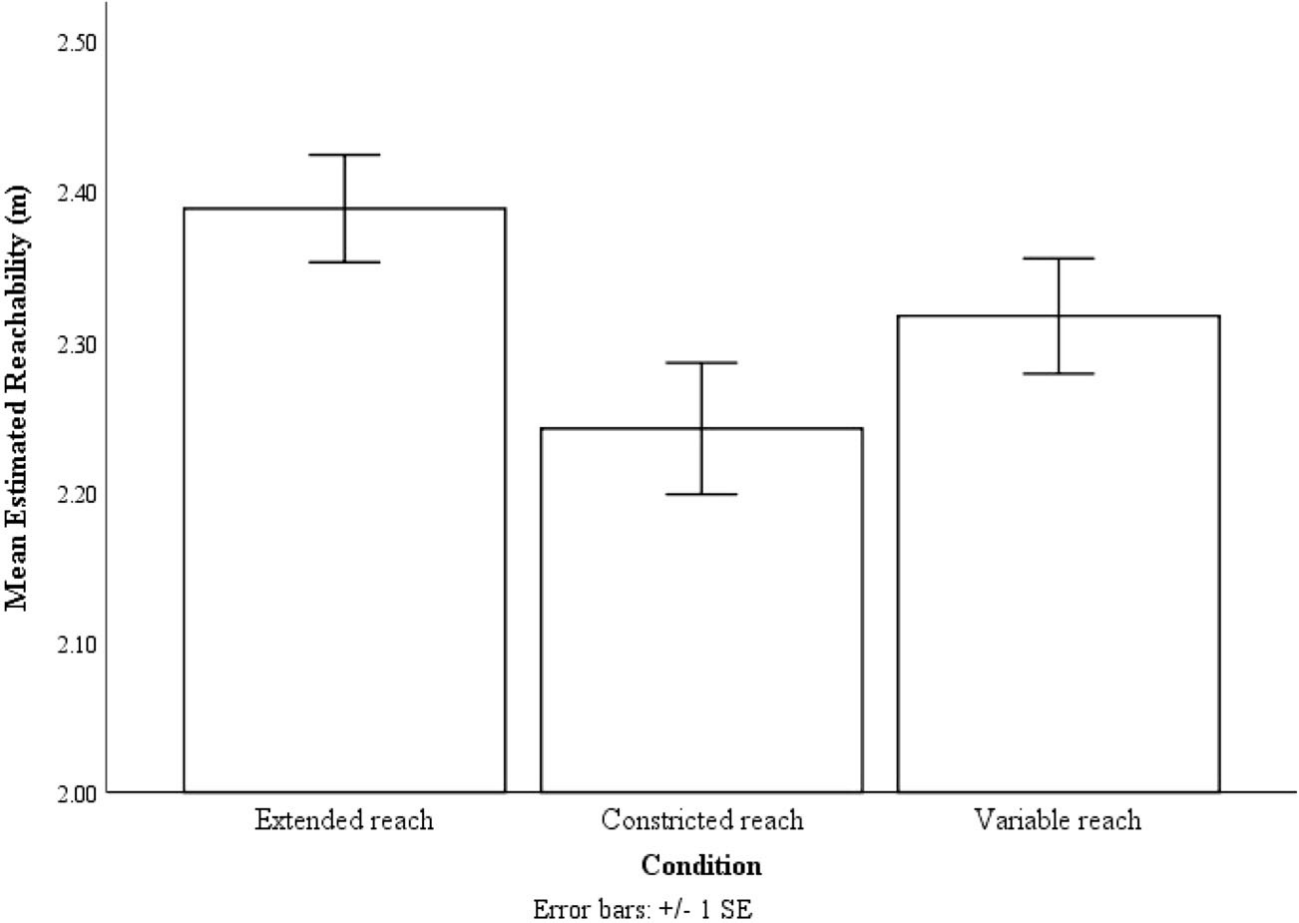
The mean estimated reachability of the three reaching conditions. Error bars are 1 ± SE calculated within-subject with the method provided by Loftus and Masson (1994)

As predicted, analysis showed effects of reaching condition on estimated reachability, F(2,40) = 14.96, p = .001, ƞp^2^ = .43. Bonferroni post hoc analysis showed that participants estimated the extent of their reach as being farther in the extended reach condition (M = 2.39 m, SE = .04 m) than in the constricted reach condition (M = 2.24 m, SE = .04 m, p < .001). They also estimated their reachability to be farther in the variable condition (M = 2.32 m, SE = .04 m, p = .04) than in the constricted reach condition. Furthermore, they have estimated their reachability to be farther in the extended reach condition than in the variable reach condition (p = .02) (see Fig. 3).

These results indicate that there was evidence for a difference between the variable and both the extended and constricted reach conditions, suggesting that participants selected a moderate-sized action boundary that was smaller than the one selected in the extended reach condition but larger than the one selected in the constricted reach condition. Had they used heuristics to determine action boundary and employ a liberal tactic, we would expect their reachability estimates to be similar to their reachability estimates in the extended reach condition. Alternatively, had participants employed a conservative tactic as a heuristic strategy, we would expect their reachability estimates to be similar to those in the constricted reach condition. Instead, we found that participants opted for a moderate-sized action boundary after experiencing the three different reaches with equal probability (i.e., equal number of trials), and this strategy is consistent with what would be expected if participants had used an average of their reaching experience to determine action boundary.

## Experiment 2

Findings from Experiment 1 demonstrated that when the perceptual-motor experience was completely random in that individuals experienced all three reaches with equal probability, individuals selected an averaged-size action boundary size that was smaller than the one selected in the extended reach condition but larger than the one selected in the constricted reach condition. Findings from Experiment 1 were consistent with what would be expected if individuals were using a weighted average of their reaching experience to determine their action boundary for reaching. In Experiment 2, we sought to investigate how individuals select their action boundary when the perceptual-motor experience is systematically weighted towards the extended arm’s reach and that they experienced the farther reach twice as often. If individuals were using a weighted average of their experience, we would expect them to favour a larger action boundary as more weight would be assigned to the larger action boundary.

## Method

### Participants

Twenty-four participants (19 Females) between 18 to 49 years of age (*M*_*age*_ = 23.67 years, *SD*_*age*_ = 7.24 years) were recruited from Lancaster University through opportunity sampling. All participants but two were right-handed. All participants had normal or corrected-to-normal vision. All participants provided informed consent. This study was approved by the ethics committee at Lancaster University.

### Stimuli and Apparatus

The experimental set-up was the same as in Experiment 1. Participants estimated their maximum reachability after being calibrated with a long virtual arm, a short virtual arm or a virtual arm that varies in size systematically.

### Procedure

The procedure was the same as in Experiment 1. In the variable reach condition of this experiment, 50% of their reaches had the extended arm's reach, 25% of the reaches had the constricted arm's reach, and 25% of their reaches had the normal arm's reach. All reaches were experienced in randomised order.

## Results

To account for the height of the building rooftop that the participants were standing on, we have subtracted 64.228 from the raw reaching estimates. A repeated-measures ANOVA was conducted with reaching condition (extended/ constricted/variable) as within-subjects variable and the estimated reachability as the dependent variable.

**Fig. 4:**
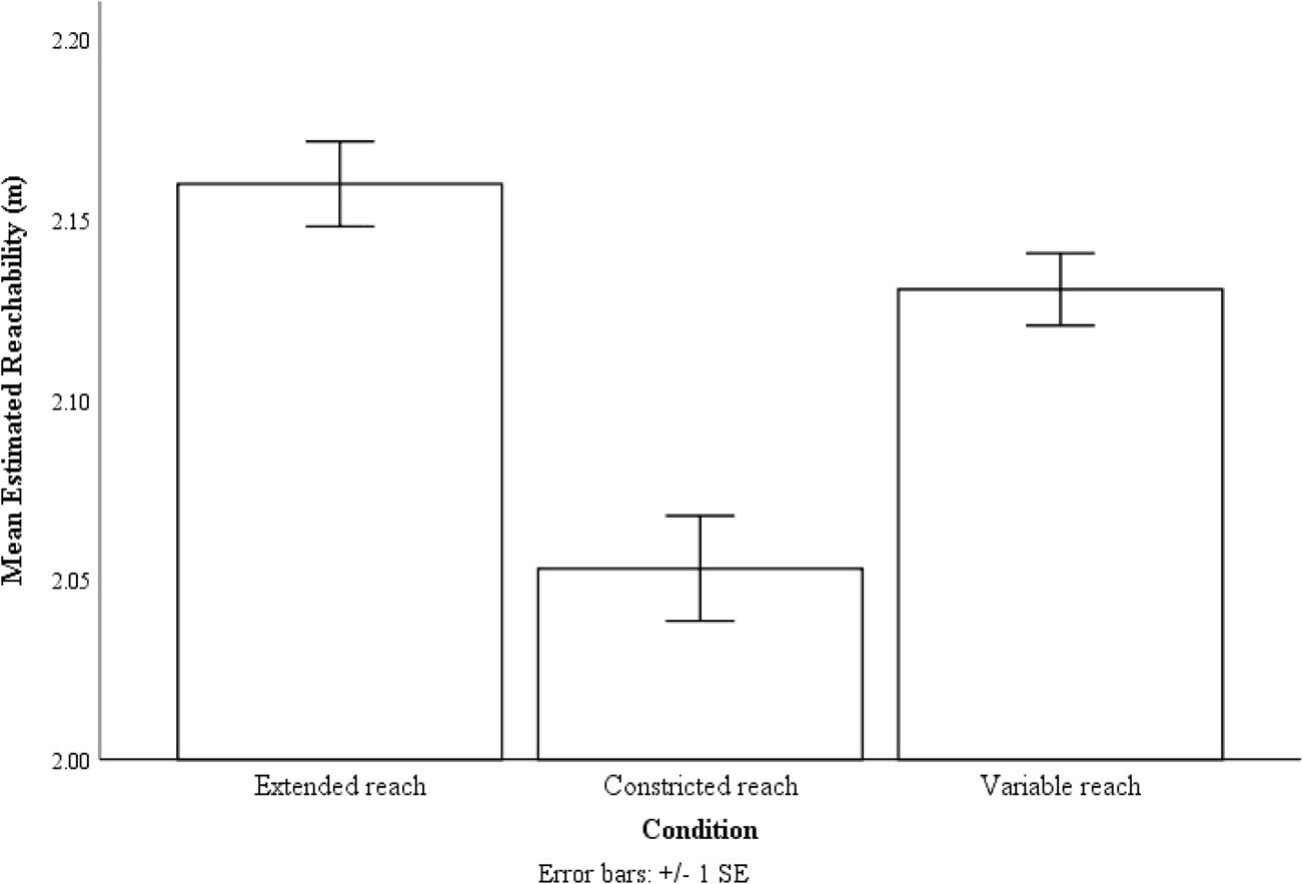
The mean estimated reachability of the three reaching conditions. Error bars are 1 ± SE calculated within-subject with the method provided by Loftus and Masson (1994)

There was a main effect of reaching condition on estimated reachability, F(2, 46) = 13.44, p < .001, ƞp^2^= .37. Participants estimated their reachability to be farther in the extended reach condition (M = 2.16 m, SE = .04 m) than in the constricted reach condition (M = 2.05 m, SE = .04 m, p = .001). They also estimated their reachability to be farther in the variable condition (M = 2.13 m, SE = .03 m, p = .01), than in the constricted reach condition (see Fig. 4). Furthermore, we found no evidence for a difference between the extended and variable reach conditions (p = .26).

These results demonstrated that the perceived reachability was affected by the type of perceptual motor variability present. Specifically, when the perceptual motor experience was systematically weighted in that participants experienced the farther reach substantially more often than other reaches, participants were more liberal with their reachability estimates than when all reaches with experienced with equal probability. Taken together with Experiment 1, these results provide further evidence that participants were sensitive to the probabilistic information associated with each arm’s reach they have experienced, and a weighted average of reaching experience was used to determine action boundaries under conditions of uncertainty.

## Experiment 3

Findings from Experiments 1 and 2 showed that individuals were sensitive to the type of perceptual-motor variability present and used a weighted average of reaching experience to determine action boundaries. However, a question remains as to whether the perceptual system takes into account the environmental context in which the action occurs and employ different strategies to determine action boundaries. Findings from Experiments 1 and 2 can be interpreted as participants recognising the costs associated with failed actions in the height related situation and therefore being more cautious with the selection of action boundaries. It is possible that in the absence of cost to making errors, individuals would opt for a more time-efficient and less deliberate method to determine action boundaries. They may adopt a single action boundary regardless of changes in probabilistic information associated with each action boundary experienced, similar to those reported in Lin et al. (2020). Hence, in Experiment 3, we sought to investigate how individuals select their action boundaries for overhead reaching in a low-risk (non-height-related) situation.

## Method

### Participants

Twenty participants (15 females) between 18 and 22 years of age (Mage = 19.15 years, SDage = 1.39 years) were recruited from Lancaster University through opportunity sampling. All participants were right-handed. All participants had normal or corrected-to-normal vision. All participants provided informed consent. This study was approved by the ethics committee at Lancaster University.

#### Stimuli and apparatus

**Fig. 5:**
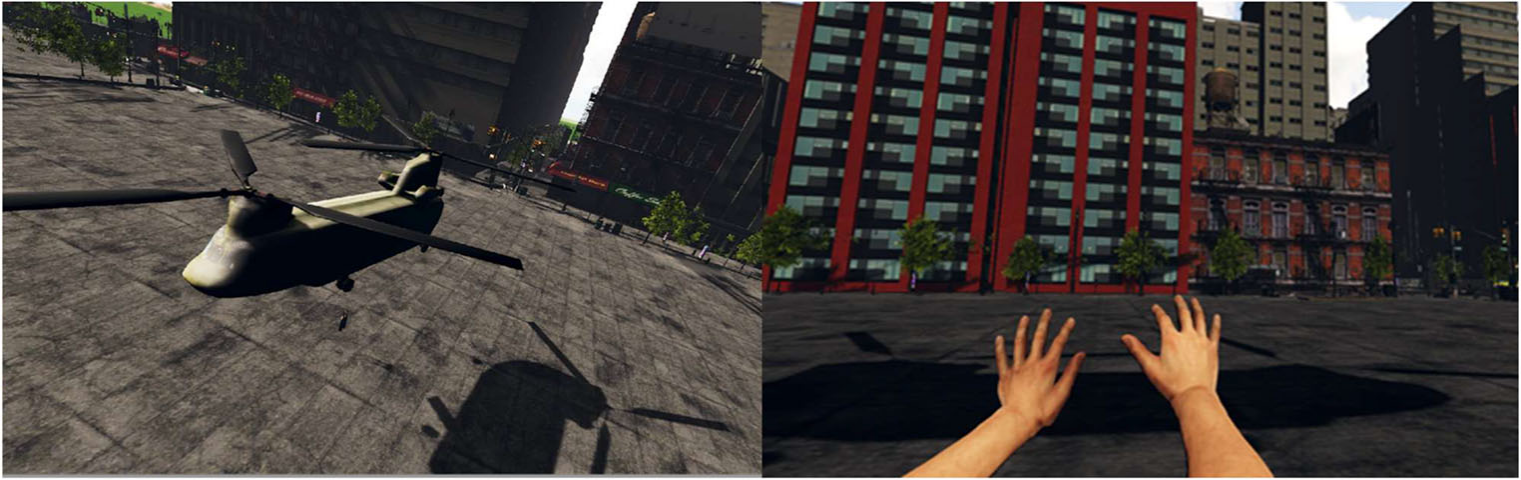
Left panel: Screenshot of the virtual environment showing the complete scene. Right panel: Image of what the participant would see from their perspective during the calibration trial

The experimental setup was similar to Experiments 1 and 2. The experimental program and environment were created using Unity 3D© Gaming Engine with the Leap Motion plugin. For the virtual environment, a 3D model of a city with skyscrapers was used. The virtual avatar was placed in a city square/plaza surrounded by trees and buildings; a 3D model of a transport chopper was placed above the avatar, with a ladder that extended from the bottom of the chopper (see Fig. 5).

#### Procedure

The procedure was the same as Experiments 1 and 2, but instead of rooftop, participants performed the calibration and estimation phase while standing on a horizontal ground plane. Participants estimated their maximum reachability after being calibrated with a long virtual arm, a short virtual arm or a virtual arm that varies in size randomly. In the variable reach condition of this study, participants experienced all three reaches with equal probability.

## Results

**Fig. 6:**
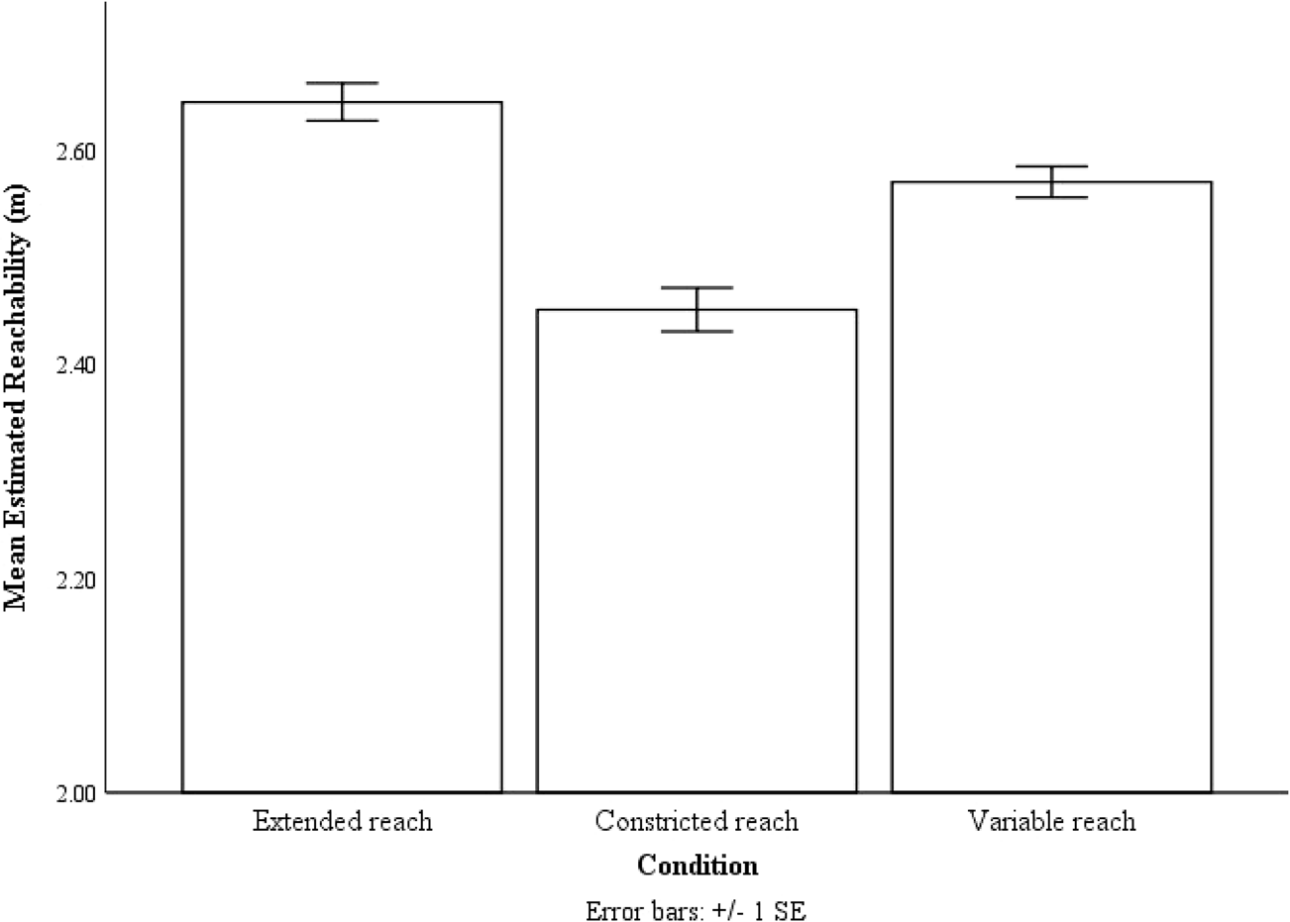
The mean estimated reachability of the three reaching conditions. Error bars are 1 ± SE calculated within-subject with the method provided by Loftus and Masson (1994)

A repeated-measures ANOVA was conducted with reaching condition (extended/ constricted/variable) as the within-subjects variable and the estimated reachability as the dependent variable. Analysis showed effects of reaching condition on estimated reachability, F(2,38) = 20.55, p < .001, ƞp^2^ = .52. Bonferroni post hoc analysis showed that participants estimated the extent of their reachability as being farther in the extended reach condition (M = 2.64 m, SE = .04 m) than in the constricted reach condition (M = 2.45 m, SE = .04 m, p < .001). They estimated their reachability to be farther in the variable condition (M = 2.57 m, SE = .04 m, p = .003) than in the constricted reach condition. Furthermore, they estimated their reachability to be farther in the extended reach condition than in the variable reach condition (p = .02) (see Fig. 6).

These results indicate that the estimates in the variable condition significantly differed from the estimates in both the extended and the constricted conditions, suggest that participants selected a moderate-sized action boundary after experiencing all three reaches with equal probability. Based on these findings, it is reasonable to postulate that participants selected their action boundary using a weighted average of their reaching experience. However, it is also possible that the perceptual system was merely adopting a moderate action boundary without taking the probabilistic information into account, as failed action in this environmental context was not associated with dangerous consequences. Hence, in order to provide more clarity, in the next experiment, we investigated whether perceived reachability was altered by more extensive experience with the farther reach.

## Experiment 4

Findings from Experiment 3 demonstrated that when the perceptual-motor experience is completely random in that individuals experienced all three reaches with equal probability, individuals selected an averaged size action boundary size that was smaller than the one selected in the extended reach condition, but larger than the one selected in the constricted reach condition. However, it remained unclear as to whether the perceptual system took into account the probabilistic information associated with each action boundary experienced, or was merely selecting a moderate-sized action boundary as an effort-reduction strategy. Thus, in Experiment 4 we investigated the effect of systematic variability on the perception of action boundaries in a non-height-related situation. If individuals were not taking probabilistic information into account, then given the absence of costs to making errors, we would not expect to see an increase in action boundary size despite having more experience with the extended arm’s reach, and action boundary selected would be similar to the action boundary selected in Experiment 3. However, if individuals were taking the probabilistic information into account and used a weighted average of reaching experience to determine action boundary, then we would expect participants to estimate their reachability liberally.

## Method

### Participants

Twenty participants (16 females) between 18 and 28 years of age (Mage = 21.65 years, SDage = 3.05 years) were recruited from Lancaster University through opportunity sampling. All participants but two were right-handed. All participants had normal or corrected- to-normal vision. All participants provided informed consent. This study was approved by the ethics committee at Lancaster University.

#### Stimuli and apparatus

The experimental set-up was the same as in Experiment 3. Participants estimated their maximum reachability after being calibrated with a long virtual arm, a short virtual arm or a virtual arm that varies in size systematically.

#### Procedure

The procedure was the same as Experiment 3. In the variable condition of this experiment, 50% of their reaches had the extended arm's reach, 25% of the reaches had the constricted arm's reach, and 25% of their reaches had the normal arm's reach. All reaches were experienced in randomised order.

## Results

A repeated-measures ANOVA was conducted with reaching condition (extended/constricted/variable) as within-subjects variable and the estimated reachability as the dependent variable.

**Fig. 7:**
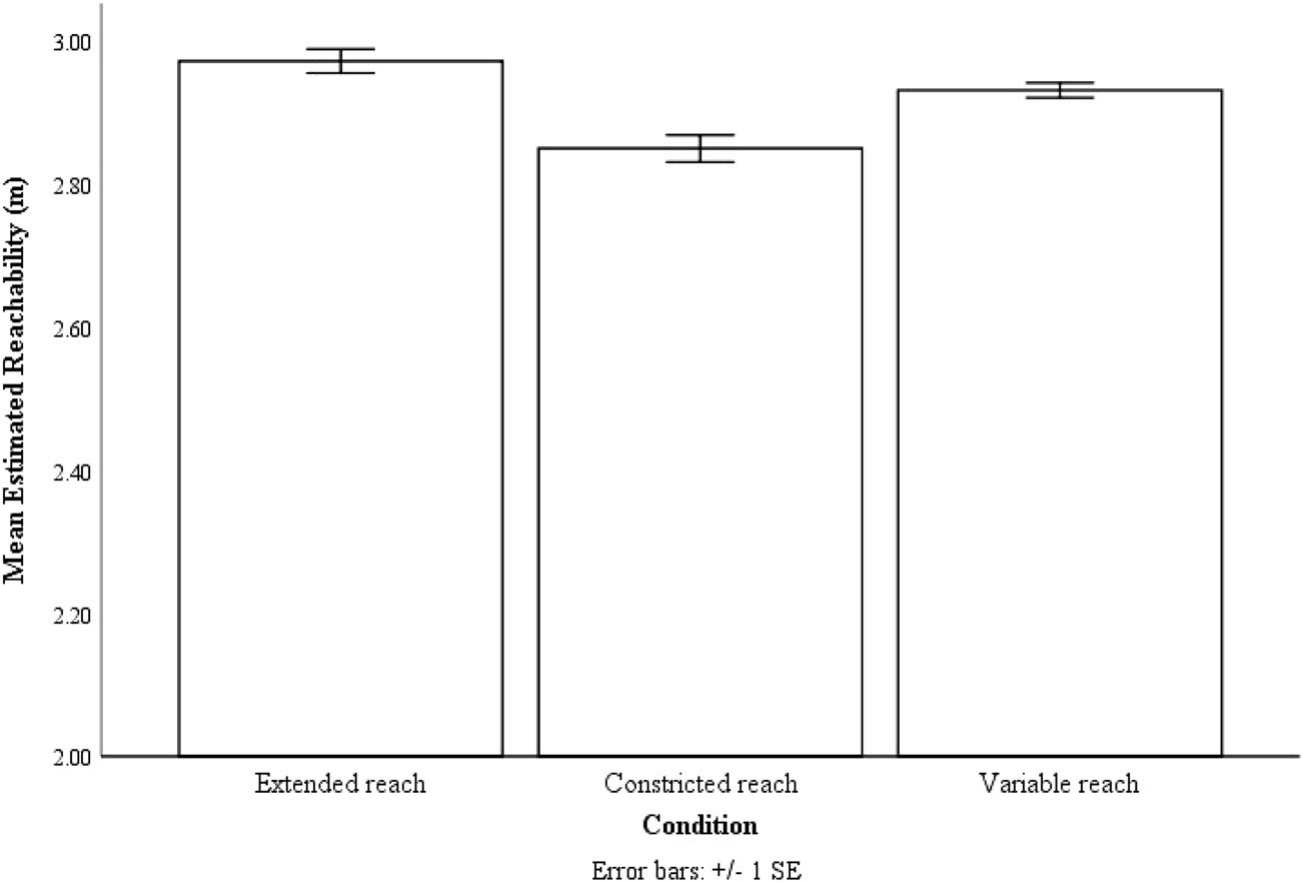
The mean estimated reachability of the three reaching conditions. Error bars are 1 ± SE calculated within-subject with the method provided by Loftus and Masson (1994)

The analysis provided Greenhouse-Geisser-corrected degrees of freedom to account for possible violations of sphericity, therefore the degrees of freedom were not always integers. As predicted, analysis showed effects of reaching condition on estimated reachability, F(1.50, 28.52) = 10.50, p < .001, ƞp^2^ = .36. Bonferroni post hoc analysis showed that participants estimated the extent of their reach as being farther in the extended reach condition (M = 2.97 m, SE = .04 m) than in the constricted reach condition (M = 2.85 m, SE = .05 m, p = .01). They also estimated their reachability to be farther in the variable condition (M = 2.93 m, SE = .04 m, p = .01) than in the constricted reach condition. However, no difference was found between the variable and extended reach condition (p =.20) (see Fig. 7).

These results demonstrated that perceived reachability was influenced by the type of variability present, and more extensive experience with the farther reach has led participants to increase their reachability estimates to a more liberal size. Additionally, these findings showed that our findings from the previous experiment was not the result of the perceptual system adopting a moderate-sized action boundary as a heuristic strategy, instead, the perceptual system was taking the probabilistic information associated with each action boundary experienced into account and used a weighted average to determine action boundary.

## Across four experiments

Findings across four experiments revealed that similar strategies were used in both height- and non-height-related contexts to determine action boundaries following the experience of perceptual motor variability. Regardless of environmental contexts, following experience reaching where their reaching length varied drastically, participants selected action boundary using a weighted average. Although environmental context did not appear to influence the strategy by which action boundaries were determined as a result of high versus low perceptual motor experience, it is possible that it has an additive effect on action boundary selection, with participants being more conservative overall (in both high and low variability conditions) with their reachability estimates in the height-related context. In order to assess the influence of environmental context on estimated reaching ability, we collapsed across Experiments 1 and 3 (random variability) and Experiments 2 and 4 (systematic variability), and analysed the combined data to get a better idea of the relationship between environmental context and perceived action boundaries.

## Across Experiments 1 and 3

We conducted a repeated-measures ANOVA with mean estimated reachability (Extended/Constricted/Variable) as within-subjects variable and the environmental context (Height-related/Non-height-related) as between-subject variable. We found an effect of reaching condition F(2,78) = 35.57, p < .001, ƞp^2^ = .48, with the mean extended reach (M =

**Fig. 8:**
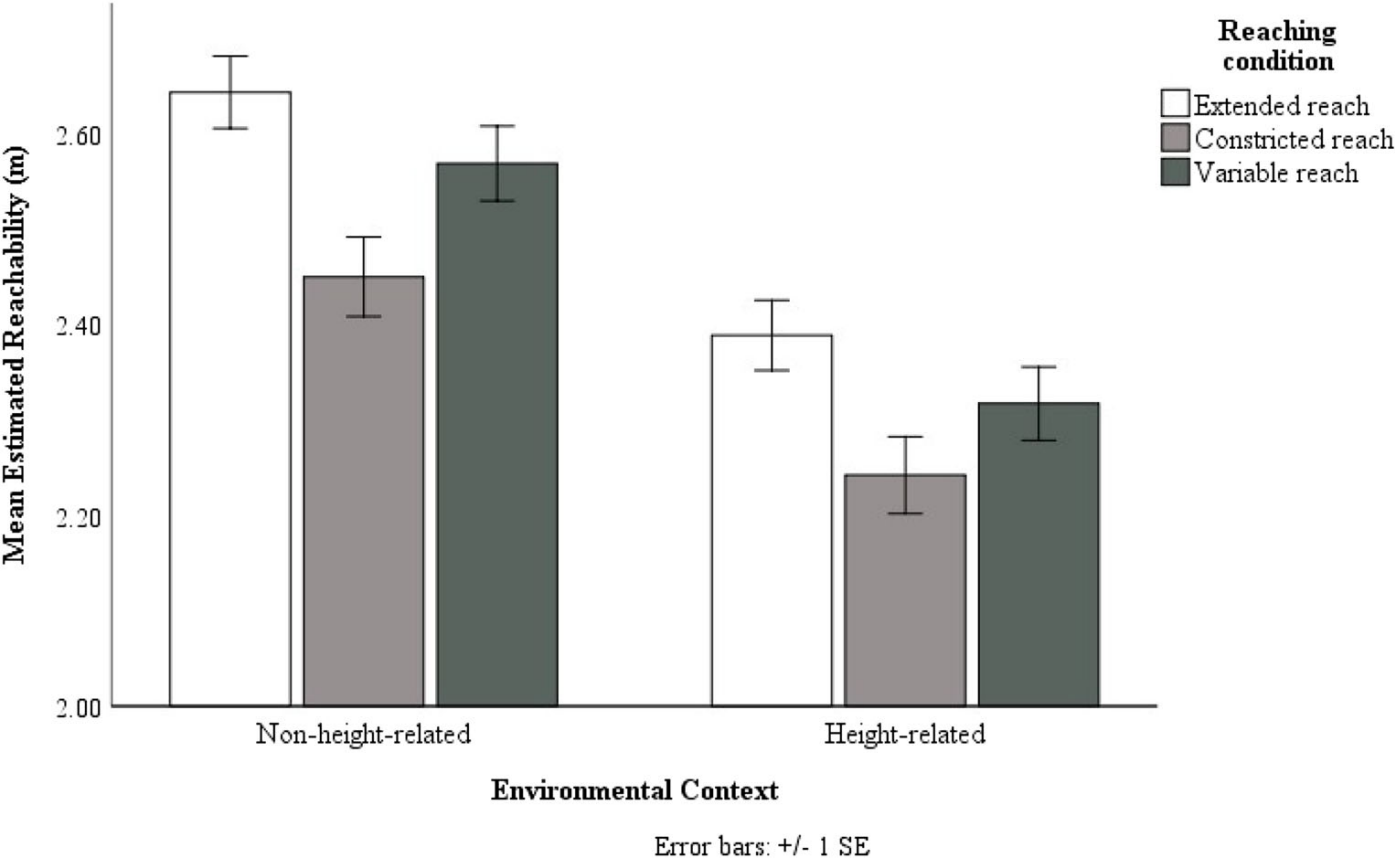
The mean estimated reachability of the three reaching conditions across the two environmental contexts. Error bars represent 1 ± SE of the mean

2.52 m, SE = .03 m) being larger than the mean constricted reach (M = 2.35 m, SE = .03 m, p <.001) and the mean variable reach (M = 2.44 m, SE = .03 m, p < .001). Furthermore, the mean variable reach was also larger than the mean constricted reach (p < .001). Analysis showed effects of environmental contexts on estimated reachability, F(1,39) = 22.60, p < .001, ƞp^2^ = .37, with reachability estimates in the non-height-related conditions (M = 2.55 m, SE = .04 m) being significantly larger than those in the height-related conditions (M = 2.32 m, SE = .04 m, p < .001) (see Fig. 8). The interaction between reaching condition and environmental context was not significant, F(2,78) = .85, p = .43, ƞp^2^ = .02). These results suggest that overall in Experiments 1 and 3 participants’ reachability estimates were more conservative in the height- related conditions than in the non-height-related conditions.

**Fig. 9:**
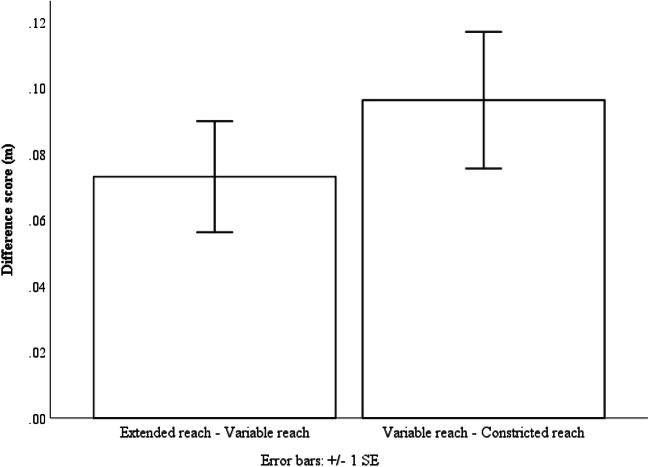
The EV and VC difference scores collapsed across Experiment 1 and 3. Error bars represent 1 ± SE of the mean

To get a better idea of the relationships between the three reaching condition, using the collapsed data, we created two difference scores for each participant. We created one difference score by subtracting the mean variable reach estimate from the mean extended reach estimate (EV) and the other by subtracting the mean constricted reach estimate from the mean variable reach estimate (VC). If participants used a weighted average to determine their action boundary in the random variability conditions, we should expect no difference between the EV and VC scores. A paired-samples t-test was conducted to compare the difference between the EV and VC scores. The t-test found no evidence for a difference between the EV scores (M = .07 m, SD = .11 m) and the VC scores (M = .07 m, SD = .13 m); t(20) = -.07, p = .94 (see Fig. 9). These findings indicate that in the random variable reach conditions participants likely used a weighted average to determine action boundary, and the action boundary size selected was in between the extended reach condition and the constricted reach condition.

## Across Experiments 2 and 4

**Fig. 10:**
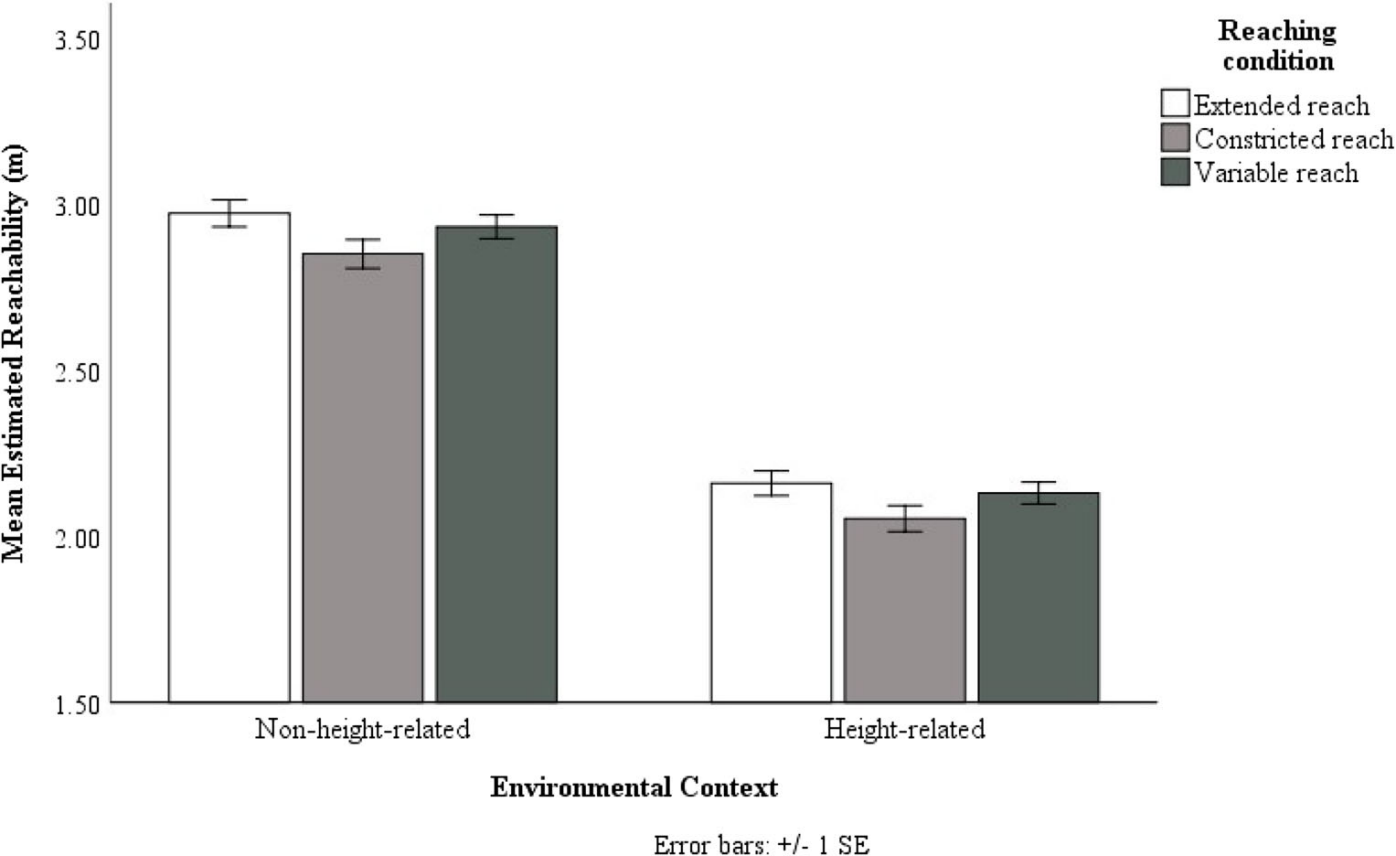
The mean estimated reachability of the three reaching conditions across the two environmental contexts. Error bars represent 1 ± SE of the mean

We conducted a repeated-measures ANOVA with mean estimated reachability (Extended/Constricted/Variable) as within-subjects variable and the environmental context (Height-related/Non-height-related) as between-subjects variable. We found an effect of reaching condition F(1.60,67.35) = 23.79, p < .001, ƞp^2^ = .36, with the mean extended reach (M = 2.57 m , SE = .03 m) being larger than the mean constricted reach (M = 2.45 m, SE = .03 m, p <.001) and the mean variable reach (M = 2.53 m, SE = .02 m, p = .03). Furthermore, the mean variable reach was larger than the mean constricted reach (p < .001). Analysis showed effects of environmental contexts on estimated reachability, F(1,42) = 254.208, p < .001, ƞp^2^ = .86, with reachability estimates in the non-height-related conditions (M = 2.92 m, SE = .04 m) being significantly larger than those in the height-related conditions (M = 2.12 m, SE = .03 m, p < .001) (see Fig. 10). The interaction between reaching condition and environmental context was not significant, F(2,84) = .11, p = .90, ƞp^2^ = .003). These results suggest that in Experiments 2 and 4, participants’ reachability estimates were more conservative in the height-related conditions than in the non-height-related conditions.

**Fig. 11:**
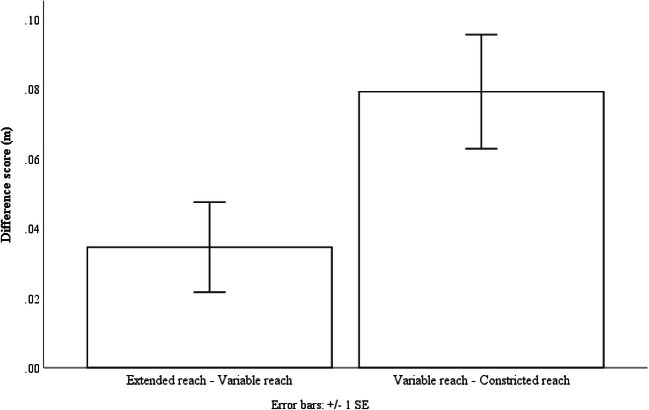
The EV and VC difference scores collapsed across Experiment 2 and 4. Error bars represent 1 ± SE of the mean

Using the collapsed data from Experiments 2 and 4, we created one difference score by subtracting the mean variable reach estimate from the mean extended reach estimate (EV) and the other by subtracting the mean constricted reach estimate from the mean variable reach estimate (VC). If participants used a weighted average to determine their action boundary in the systematic variability conditions, we should expect a difference between the EV and VC scores. A paired-samples t-test was conducted to compare the difference between the EV and VC scores. We found an effect of difference scores with the EV scores (M = .03, SD = .09) being smaller than the VC (M = .08, SD = .11); t(43) = -2.08, p = .04, indicating that estimates in the systematic variable conditions were closer to the extended reach estimates than the constricted reach estimates (see Fig. 11). Participants used a weighted average to determine their action boundaries and were estimating liberally in the systematic variable reach conditions.

Taken together, these findings suggest that while the similar strategies were used in both height- and non-height-related contexts to determine action boundary, participants were significantly more conservative with their reachability estimates in the height-related context than they were in the non-height-related context.

## Discussion

Recent studies using a similar paradigm have investigated the influence of perceptual- motor variability on the perception of action boundaries for horizontal reaching, and found that individuals tended towards liberal estimates of their reachability in the event of perceptual motor variability. However, from these results, one could not determine whether the perceptual system utilises the same strategy to determine action boundaries for all types of reaches and different environmental contexts. Hence, in this set of studies, we examined the effect of different types of perceptual-motor variability and environmental contexts on the perception of action boundaries for overhead reaching. Participants were asked to estimate the maximum vertical reachability following calibration to a long virtual arm, a short virtual arm or a virtual arm that varied in size either randomly, or systematically weighted in that the long virtual arm was experienced twice as often. We also contrasted participants’ recalibration of action boundaries following changes in their action capabilities in two situations: a height-related situation (Experiments 1 and 2) and a non-height-related situation (Experiments 3 and 4). The perceived penalty for error was presumably more severe in the height-related situation, which enabled us to examine whether participants take into account the context in which the action occurs with the selection of action boundaries in the event of perceptual-motor variability.

We replicated the effect of perceptual motor experience on perceived reachability reported by Lin et al. (2020). Our findings demonstrated that participants were sensitive to changes in their action capabilities, and their reachability judgements were consistent with their reaching experience during calibration phases. In all four experiments, participants consistently estimated their reachability in the extended reach condition to be farther than in the constricted reach condition. This finding provides further evidence that manipulation of perceptual motor feedback could influence perceived action boundaries, and perceptual motor recalibration could occur after a brief exposure in virtual environments.

Experiments 1 and 3 demonstrated that when faced with random variability in their reaching experience and participants experienced all three reaches with equal probability, participants subsequently reported their reachability to resemble the mean. Experiments 2 and 4 indicated that when reaching experiences were greatly weighted towards the extended arm’s reach, participants opted for a more liberal, larger action boundary for reaching. Taken together, results from the four experiments indicated that participants were sensitive to the probabilistic information associated with different arms’ reach they have experienced during the calibration phase, and used a weighted average of reaching experience to determine their action boundary under conditions of uncertainty.

Interestingly, our findings reveal that similar strategies were used to determine action boundaries following perceptual motor variability in both environmental contexts. Had participants employ different strategies depending on the environmental context in which the action occurred, given the absence of perceived penalty/negative consequences associated with a failed action in the non-height related situation, we would expect participants to be less deliberate with their reachability estimates. Instead, participants used a weighted average to determine action boundary following variability experience in both contexts, and participants incorporated probabilistic information associated with the reach lengths they experienced during the calibration phases into their subsequent action boundary judgements.

It was possible that participants did not perceive any negative consequences associated with failed reaching in the virtual environment, which could account for the similar pattern of results observed in both environmental contexts. In order to conclude that environmental context had no influence on the strategy by which action boundaries are determined, we collapsed across Experiments 1 and 3, as well as Experiments 2 and 4. Results from the cross-experiment analyses showed that participants were more conservative with their reachability estimates overall in the height-related context. These findings suggest that while similar strategies were used to determine action boundaries relative to perceptual motor variability in both contexts; environmental context had an additive effect on participants’ action boundary selection, with participants being more conservative with their reachability estimates across all reaching conditions in the height-related context.

Our findings here differ from those in Lin et al. (2020) in that they showed that individuals were quite liberal in their approximations of their action boundaries, whereas here we found that individuals have chosen the medium action boundary. Hence, we can reasonably postulate that the strategy in which the perceptual system employed to determine action boundary in the event of perceptual motor variability is action specific (i.e., type of reach) rather than context specific. This presumption may seem counterintuitive, however, if action boundary determination is context specific, then the strategy by which the action boundary for overhead reaching is determined would be generalised to all actions performed in the same situation (e.g., similar strategy would be employed to determine action boundary for horizontal reaching and jumping in the same situation). While this could be a more efficient approach, it is also less behaviourally adaptive, because different actions have different associated consequences and their respective costs and benefits. Employing a context- specific blanket approach to determine action boundary would not be flexible enough to account for all possible actions and their associated consequences. Although our results showed that environmental context has an additive effect on participants’ action boundary selection, we found no evidence for a context-specific effect on the strategy used by the perceptual system to determine action boundary. We are aware that we only assessed two different contexts (even though they were specifically chosen because we expected a context effect for these different contexts). It would be premature to conclude that context has no influence on the strategy in which action boundaries are determined under conditions of perceptual-motor variability. It is possible that in addition to the consequence and costs-benefits ratio of a particular action, the perceptual system may employ different strategies to determine action boundary to meet the demands of the specific situation for various other actions. Future research could expand on this further and examine the influence of different environmental contexts as well as actions on action boundary selection.

One possible interpretation for our current findings in the context of previous findings is that not all actions are important enough to warrant spending the time and effort to integrate probabilistic information and/or to generate optimal solutions. However, for some actions, it is worth the time and effort to determine the optimal solution, especially when an erroneous motor decision (or selection of inappropriate action boundary) could lead to negative consequences. In the case of overhead reaching, selecting an inappropriate may result in loss of balance and falling. Hence, a better strategy would be to forgo short term gains in efficiency for more deliberate and careful evaluation (Beach & Mitchell, 1978; Glöckner, 2008). Thus, the perceptual system would possibly behave in ways that mimic a weighted average for more than just ‘good enough’ solutions in situations where a failed attempt at an action could result in harm. Although heuristics generally provide sufficient solutions for certain actions’ boundaries, other more dangerous actions’ boundaries situations may exist in which it would be a non-adaptive strategy for human ancestors to disregard uncertainties and/ or probabilistic information. By using different approaches for different actions on an ad hoc basis to determine action boundaries, the perceptual system could maximise the efficiency of information processing in the event of perceptual motor uncertainties, while minimising the exposure to potentially dangerous situations and aversive consequences.

Similarly, our findings indicate that participants favoured a more conservative-sized action boundary for overhead reaching than for horizontal reaching as reported in Lin et al. (2020), in which participants demonstrated a tendency for a liberal estimates of their horizontal reachability regardless of whether they have experienced all three arms’ reach with equal probability, or whether their reaching experience was greatly weighted towards the constricted or the extended arm’s reach. This difference could also be attributed to the increased postural demand required by the reaching task in the present study. In Lin et al. (2020), participants were asked to estimate their reachability of one arm for horizontal objects while seated. In the present study, participants had to extend both arms upwards while standing upright with both feet on the ground, which led to reduced postural control and increased postural sway. Thus, when faced with inconsistency in the perceptual motor feedback, selecting a more conservative action boundary could be an indication of the presence of a larger safety margin. Additionally, these results resonate with findings reported in the literature suggest that there is a reduction in the magnitude of overestimation or even underestimation in perceived reachability for reaching tasks that required greater postural stability demands (Carello et al., 1989; Gabbard et al., 2007; Robinovitch, 1998). Hence, selecting action boundary using a weighted average for overhead reaching would prevent individuals from executing reaches that would jeopardise their balance and reduce the exposure to potentially adverse consequences.

In summary, the present studies extended findings from previous studies that examined the effect of perceptual motor variability on perceived action boundaries for reaching. Our findings demonstrate that the perceptual systems utilised similar strategies to determine action boundaries in both height- and non-height-related contexts, and participants used a weighted average of their reaching experience to determine action boundaries for overhead reaching under conditions of perceptual-motor variability.

### Acknowledgements and Funding Information

This work was supported by the Economic and Social Research Council (ESRC) North West

Doctoral Training Centre (NWDTC) [Grant number ES/J500094/1]
